# Efficacy and safety of vortioxetine in cardiological patients

**DOI:** 10.1192/j.eurpsy.2025.862

**Published:** 2025-08-26

**Authors:** A. Schelchkova, J. Barylnik, D. Samoylova

**Affiliations:** 1 Saratov State Medical University, Saratov, Russian Federation

## Abstract

**Introduction:**

The most important conditions for the using of antidepressants in cardiological practice are good tolerability, minimal interaction with other drugs, and the absence of a toxic effect. All of the above qualities among antidepressants have a multimodal antidepressant – vortioxetine.

**Objectives:**

Evaluation of the efficacy and safety of vortioxetine in the treatment of concomitant depressive disorders in cardiological patients.

**Methods:**

37 patients aged 45-66 years who were admitted for inpatient treatment at the Regional Clinical Cardiology Dispensary with a diagnosis of “coronary heart disease, Angina pectoris II-III FC” were examined. Upon the hospitalization, they were consulted by a psychiatrist. During the consultation, the patients were diagnosed with a Major depressive disorder, single episode, moderate (F 32.1 according to ICD – 10). Therapy of depressive disorder was carried out in average therapeutic doses with vortioxetine for 8 weeks. To evaluate the efficacy, scales and questionnaires were used: HADS, PDQ–20, CGI–C, SF–36. Statistical processing of the material was carried out using a package of applied statistical programs: Excel, Word (version 2108).

**Results:**

Analysis of the results of assessing the severity of depressive and anxiety manifestations on the HADS scale and the level of cognitive functioning using the PDQ questionnaire allowed us to state that at the end of 8 weeks of therapy with vortioxetine, 89% of patients showed significant clinical improvement. The positive dynamics of patients is confirmed by data on the CGI–C scale. The answers to the SF–36 questionnaire indicate an improvement in the life quality indicators. Statistically significant changes were observed in the parameters “Physical condition”, “Mental health” and “Vital activity”.

The tolerability of vortioxetine in all patients was good, side effects in 13.5% of the subjects in the form of dizziness, nausea and daytime drowsiness were reduced within 1 week.

There were no significant changes in the biochemical parameters of blood, coagulogram, electrolyte composition. When assessing the indicators of blood pressure, heart rate, there was a significant improvement in indicators by the end of the research. In 8 weeks, the frequency of angina attacks and arrhythmias significantly was decreased.

**Conclusions:**

When prescribing vortioxetine to patients with cardiological pathology for 8 weeks, there was distinct positive dynamics both in terms of indicators of the cardiovascular system, and in the emotional sphere, cognitive and social functioning.

**Disclosure of Interest:**

None Declared

